# Quantities and units for electrophoresis in the clinical laboratory

**DOI:** 10.1155/S1463924692000014

**Published:** 1992

**Authors:** G. Férard

**Affiliations:** 7 rue des Jardins Fleuris Strasbourg F67000 France

## Abstract

Electrophoretic techniques have been developed and refined over decades, and are now widely used in clinical laboratories. For example, electrophoresis is routinely used to separate many different components, including proteins, lipoproteins, and isoenzymes. More recently, the applications of molecular biology in diagnosis have increased the use of electrophoresis to separate DNA components in the clinical laboratory. Various kinds of quantities are used for the description of separation procedures. It is the purpose of this document to provide manufacturers and users of electrophoretic techniques with a list of relevant quantities and units consistent with the International System of Units (SI) and standards of the International Organization for Standardization (ISO).


Journal of Automatic Chemistry, Vol. 14, No. 1 (January–February 1992), pp. 1–4

### Quantities and units for electrophoresis in the clinical laboratory

**G. Férard for the International Union of Pure and Applied Chemistry, Clinical Chemistry Division, Commission on Quantities and Units in Clinical Chemistry and International Federation of Clinical Chemistry, Scientific Division, Committee on Quantities and Units**

*Comments on this paper and requests for offprints should be sent to Dr G. Férard, 7 rue des Jardins Fleuris, Strasbourg, F67000, France*

*Electrophoretic techniques have been developed and refined over decades, and are now widely used in clinical laboratories. For example, electrophoresis is routinely used to separate many different components, including proteins, lipoproteins, and isoenzymes. More recently, the applications of molecular biology in diagnosis have increased the use of electrophoresis to separate DNA components in the clinical laboratory. Various kinds of quantities are used for the description of separation procedures. It is the purpose of this document to provide manufacturers and users of electrophoretic techniques with a list of relevant quantities and units consistent with the International System of Units (SI) and standards of the International Organization for Standardization (ISO).*

### General definitions

A *quantity* is a measurable, real property, physical or chemical, of a specified system. It can be expressed as the production of a *numerical value* and a *unit*:

Quantity = numerical value·unit.

By convention, seven SI *base kind-of-quantities* have been defined, each with its own independent dimension. An SI *base unit* is defined for each of them.

A *kind-of-quantity* is an abstract concept of a measurable property, common to a number of real phenomena. Examples are length and electric current.

| *Base kind-of-quantity* | | *SI base unit* | |
|---|---|---|---|
| *Name* | *Symbol* | *Name* | *Symbol* |
| Length | $l$ | metre | m |
| Mass | $m$ | kilogram | kg |
| Time | $t$ | second | s |
| Electric current | $I$ | ampere | A |
| Thermodynamic temperature | $T$ | kelvin | K |
| Amount of substance | $n$ | mole | mol |
| Luminous intensity | $I_V$ | candela | cd |

All other quantities are *derived kind-of-quantitites*, defined algebraically from base kind-of-quantitites. *Derived units* are defined analogously [1–7].

### Alphabetic list of kind-of-quantities and units for electrophoresis

| *Name of kind-of-quantity* | *Symbol* | *SI unit* |
|---|---|---|
| *Charge number (of an ion B)*<br>Charge number of an ion equals the *electric charge of the ion* divided by the elementary charge of a proton. This definition applies to specified entities B:<br>$z_B = Q_B/e$.<br>The value is positive for cations and negative for anions.<br>The symbol $z$ is also used for *net electric charge* of a particle. | $z$ | 1 |
| *Electric charge*<br>Electric charge (positive or negative) equals the integral of *electric current* over time.<br>It is a synonym for the amount of electricity.<br>The name of the SI unit for electric charge is coulomb<br>(C = s A). | $Q$ | C |
| *Electric current*<br>Electric current is a base kind-of-quantity.<br>The symbol $I$ may carry one of the subscripts: a for anodic, c for cathodic, e or o for exchange, or l for limiting: $I_a$ is the anodic partial current (the anode is the electrode which is positively charged in comparison to the cathode).<br>Note that $I$ is also the symbol for *ionic strength*. | $I$ | A |
| *Electric current areic density*<br>Electric current areic density is the electric current divided by the area:<br>$J = I/A$.<br>Note that $J$ is also the symbol for flux.<br>This kind-of-quantity was named electric current (area) density [7]. | $j, J$ | A m$^{-2}$ |

© IUPAC/IFCC 1992

*Electric field strength* $E$ V $m^{-1}$
Electric field strength equals the *force* exerted by an electric field on an electric point charge divided by the *electric charge*:
$E = F/Q$.
Note that in electrophoresis $F$ is counteracted by a counter-force $F'$.
This counter-force is expressed by Stokes' law:
$F' = 6\pi r\eta v$ where $r$ is the ionic radius of the particle, $\eta$ the *viscosity* of the electrophoresis medium, and $v$ the *velocity* of the particle.
Electric field strength is a vector quantity.

*Electric potential* $V$ V
Electric potential is for electrostatic fields a scalar quantity, the gradient of which, with reversed sign, is equal to the *electric field strength.*
The name of the SI unit for electric potential is volt ($V = J\ C^{-1} = J\ s^{-1}\ A^{-1}$).

*Electric potential difference* $U$ or $\Delta V$ V
Electric potential difference is the difference in electric potential:
$U = V_2 - V_1$.
The name of the SI unit for the electric potential difference is volt.
The commonly used word voltage is not recommended.

*Electromotive force* $E$ V
Electromotive force of a source is the energy supplied by the source divided by the *electric charge* transported through the source.
The abbreviation EMF is not recommended.

*Electrokinetic potential* $\zeta$ V
Electrokinetic potential is the *electric potential difference* between the fixed charges of the immobile support and the diffuse charge in the solution. It is also called zeta potential.

*Electrophoretic mobility* $\mu$ $m^2\ s^{-1}\ v^{-1}$
Electrophoretic mobility is the observed rate of migration of a component ($v$) divided by *electric field strength* ($E$) in a given medium (see Appendix).
The symbol $\mu$ applies to entities B. It is also used for *friction coefficient.*
Note 1: Mobilities are sometimes expressed with a negative sign, because migration of the solutes or particles generally occurs in the direction opposite to the electrophoretic field (which is taken as reference for the direction).
Note 2: In a solid support medium, only apparent values can be determined.

*Elementary charge* $e$ C
Elementary charge is the *electric charge* of the proton.

*Energy* $Q$ J
The name of the SI unit for energy is joule ($J = m^2\ kg\ s^{-2}$).
Note: $Q$ is also the symbol for electric charge.

*Force* $F$ N
The resultant force acting on a body is equal to the rate of change of the momentum of the body. The name of the SI unit for force is Newton
($N = m\ kg\ s^{-2}$).
Force is a vector quantity.
Note: $F$ is also the symbol for Faraday constant.

*Friction coefficient* $\mu$ 1
Friction coefficient is the ratio of frictional forces to normal *forces* for a sliding body.
Note that $\mu$ is also the symbol for *electrophoretic mobility.*

*Ionic strength (of a solution)* $I$, $I_C$ mol $m^{-3}$
Ionic strength of a solution is half the sum of the products of the ionic charge squared and substance concentration of each ion:
$I_c = 1/2\Sigma(z_B{}^2 c_B)$.
Ionic strength may be expressed on a molality basis in mol $kg^{-1}$, using the symbol $I$ or $I_m$.

*Isoelectric point (of an elementary entity)* (pl) 1
The isoelectric point of an elementary entity is the pH at which the *net electric charge* of the entity is zero.
pl is a commonly used symbol for this kind-of-quantity. It should be replaced by pH(I)

because it is a pH determined under that particular condition.

*Isoionic point (of an elementary entity)* — 1

The isoionic point of an elementary entity is the pH value at which the *net electric charge* of the entity in pure water equals zero.

*Molar mass (of a component B)* — $M$ — kg mol$^{-1}$

Molar mass of a component B is the mass of the component divided by its amount of substance:
$M_B = m/n_B$.

*Net electric charge (of a particle)* — $z$ — 1

Net electric charge of a particle equals the algebraic sum of the charges present at the surface of the particle.
The symbol $z$ is also used for charge number of an ion.

*pH gradient* — m$^{-1}$

pH gradient is the differential change of pH with distance (dpH/d$l$).
It is a vector quantity.

*Rate of migration* — $v$ — m s$^{-1}$

Rate of migration is the distance of migration divided by time.
The rate of migration is sometimes called velocity of migration.
Note: $v$ is also the symbol for velocity.

*Temperature (thermodynamic)* — $T$ — K

*Time* — $t$ — s

*Velocity (of migration)* — $v$ — m s$^{-1}$

Velocity of migration is the distance travelled divided by time of travel:
$v = \mathrm{d}l/\mathrm{d}t$.
Velocity is a vector quantity.

*Viscosity, dynamic* — $\eta$ — Pa s = kg m$^{-1}$ s$^{-1}$

Viscosity is the constant proportionality for shear stress, $\tau_{xz}$ in a fluid moving with a velocity gradient, d$v_x$/d$z$, perpendicular to the plane of shear:
$\tau_{xz} = \eta \mathrm{d}v_x/\mathrm{d}z$.
This definition applies to laminar flow for which $v_z = 0$.

*Volume flow rate* — $q_v$, $V$ — m$^3$s$^{-1}$

Flow is the rate at which volume crosses a surface. Mass flow rate ($qm$) and substance flow rate ($qn$) may be defined analogously.
The subscripts $v$, $m$ and $n$ indicate that volume, mass and amount of substance are quantities in the numerator.

*Volume flux* — $j_v$ — m s$^{-1}$

Volume flux is the *volume flow rate* divided by the area:
$J_v = q_v/A$.
Mass flux and substance flux may be defined analogously.

### Appendix

*Example of calculation: electrophoretic mobility (μ)*

The electrophoretic mobility of albumin under specified conditions (pH, ionic strength, nature of support medium...) may be calculated as follows:

$$\mu_{\text{Albumin}} = \frac{d \cdot l}{t \cdot U}.$$

Suppose that albumin has travelled 25 mm ($d$) on a cellulose strip with a 100 mm distance ($l$) between the anodic and cathodic side of the strip in 1 h ($t$) at a potential difference of 250 V ($U$)

$$\mu_{\text{Albumin}} = \frac{(215\ \text{mm})(100\ \text{mm})}{(1\ \text{h})\quad(250\ \text{V})} = \frac{(25\ 10^{-3}\ \text{m})(100\ 10^{-3}\ \text{m})}{(3600\ \text{s})\quad(250\ \text{V})} = 0.0028\ \text{mm}^2\text{s}^{-1}\ \text{V}^{-1} \text{ or } 2.8\ 10^{-9}\ \text{m}^2\ \text{s}^{-1}\ \text{V}^{-1}.$$

*Greek letter symbols*

| | | |
|---|---|---|
| Zeta | $\zeta$ | electrokinetic potential. |
| Eta | $\eta$ | viscosity. |
| Mu | $\mu$ | electrophoretic mobility and friction coefficient. |
| Tau | $\tau$ | shear stress. |
| Sigma | $\Sigma$ | summation sign. |

### References

1. International Union of Pure and Applied Chemistry. Commission on Quantities and Units in Clinical Chemistry and International Federation of Clinical Chemistry. Expert Panel on Quantities and Units in Clinical Chemistry. DYBKAER, R., Approved recommendation (1978). Quantities and Units in Clinical Chemistry. *Clinica Chimica Acta,* **96** (1979), 157F.
2. International Union of Pure and Applied Chemistry. Physical Chemistry Division. MILLS, I., CVITAS, T., HOMANN, K., KALLAY, N. and KUCHITSU, K. *Quantities, Units and Symbols in Physical Chemistry* (Blackwell Scientific Publications, Oxford, 1988).
3. *Quantities and Units – Part 3: Mechanic* (ISO/DIS, 1990), 31.

4. *Quantities and Units – Part 5: Electricity and Magnetism* (ISO/DIS, 1990), 31.
5. *Quantities and Units – Part 8: Physical Chemistry and Molecular Physics* (ISO/DIS, 1990), 31.
6. International Union of Pure and Applied Chemistry. Commission on Quantities and Units in Clinical Chemistry and International Federation of Clinical Chemistry. Scientific Committee, Analytical Section Expert Panel on pH and Blood Gases. Siggaard-Andersen, O., Durst, R. and Maas, A. H. J., Approved Recommendations (1983), Physicochemical quantities and units in clinical chemistry with special emphasis on activities and activity coefficients. *Pure and Applied Chemistry,* **56** (1984), 567.
7. International Union of Pure and Applied Chemistry. Commission on Quantities and Units in Clinical Chemistry and International Federation of Clinical Chemistry. Scientific Committee, Nomenclature of Derived Quantitites. Rigg, J. C., Visser, B. F. and Lehmann, H. P., Recommendations 1991, *Pure and Applied Chemistry,* **63** (1991), 1307.

